# Psychological impact of COVID-19 Pandemic on dentists

**DOI:** 10.4314/ahs.v23i1.6

**Published:** 2023-03

**Authors:** Bhagyashree Patil, Sannidhya Mishra, Sanjna Ramesh, Suyog Savant

**Affiliations:** 1 Dental Surgeon, Bharati Vidyapeeth Dental College, Navi Mumbai, Maharashtra, India; 2 Department of Public Health Dentistry, Bharati Vidyapeeth Dental College, Navi Mumbai, Maharashtra, India

**Keywords:** Psychological aspect, dentist, mental health

## Abstract

**Background:**

The Covid-19 pandemic seems to have an incessant out-turn on the people in every field in some or the other way. It has been reported that maximum number of deaths in the countries during this pandemic are caused due to a term called death anxiety or phobia. There are certain parameters such as anxiety, apprehension, depression which if influence a person can alter one's well-being.

**Objective:**

The steadfast intent of this review article is to narrate the psychological impact of this pandemic on dentists. The eloquence and emergence of this topic will alarm all the medicos and paramedics to have a check on this scenario.

**Methods:**

The article consists of detailed study from several articles from PubMed publications. Articles written only in English language were referred. Various keywords such as “Covid-19 pandemic” or “Psychological Impact” were used.

**Results:**

The Covid-19 Pandemic has adversely affected all of us physically as well as psychologically. This article signifies the psychological impact of this pandemic on dentists.

**Conclusion:**

The current studies that are carried out till date show an extensive impact on the psychology of the dental professionals. The following review article elaborates the importance of the same.

## Introduction

Governments in many parts of this globe are busy in protecting humanity from the horrors of Covid-19 virus that emanated in China in the Wuhan city in December 2019^6^,^7^. All over the world, people suffered a lot in terms of health and welfare due to the spread of this virus. Although, the patient's awareness was rising to larger challenges, the Government agencies are forcing for the programme of vaccination supported by companies like Pfizer, Covishield, Covaxin etc. In major cities, because of large population there are hurdles for implementation too, still the programmes have yielded a sense of satisfaction for the people that government is doing something to safeguard the health of common population.

There are many ill effects due to this spread of covid. Many people died and families have devastated. The education system has collapsed to a certain extent, the unemployment is steeply rising. The economies of many countries have gone down. The covid virus has brought misery to humankind.

The medical noble professionals are at the forefront of this battle against Covid. Many doctors are getting the infection and are struggling for their survival. The aim is to help humanity in supporting the lifelines such as oxygen supply, remedial medicines, as well as creating necessary will power for survival. Dentists have to deal directly with the hazards of Covid ill effects and get themselves the fear of anxiety, suffering and death. The present article focuses the plight of dental practitioners and stresses the need of boosting their confidence in tackling the fear. In such situation, other remedies or means such as yoga, clean hygiene, cleaner climate and help from all health institutions can raise the psychological immunity in respect of patients as well as of doctors.

Basically Covid-19 virus is a potential respiratory disease that can cause acute respiratory tract infection. It can be transmitted from surface-to-surface contact. In the field of dentistry, it is all the way riskier due to the aerosol present in the atmosphere. It can spread through saliva, hands, and nasal droplets. The virus once enters the person's body by any means stays in the body for 4-14 days^20^. The circumstances of dental practitioners are presented and discussed in this article.

Dentistry is known as a profession filled with situations that can put the dentists in risk. Dentists already are posed to the threat of viruses like HIV, Hepatitis B & C, rubella, mumps, measles etc. Recent researches have suggested there are several oral manifestations of Covid-19 that have become prevalent for a considerable period. These oral manifestations were papilla inflammation of Wharton's duct to plaques on the soft tissues and tongue. Lesions, drug reactions and other complications caused by prolonged intubation were also being included in this categorization^8^. Hence the arrival of such a virus makes it even scarier for them. Apart from the novel corona virus infection impact, psychologically there is always a constant feeling of getting their family infected because of them doing patients. Hence considering all the aspects, this pandemic period has brought a real time experience of tremendous stress and mental pressure. This pandemic has not only hampered the routine flow of the patients in the clinics which has been drastically reduced due to the psychological fear of getting infected but also the working system of the clinics. In the post pandemic period or now also when the dentists are treating emergency cases there is absolute necessity of preparedness amongst the dentists psychologically as well as in concern with the protective PPE kits, masks. The aerosol producing treatments are the most dangerous factor and which is the sole and the ultimate reason of getting infected from the corona virus^7^. Also, not only the fear of catching the novel corona virus infection the dentists are also worried for the future of the profession which might get adversely affected. Running the family in financial terms during this pandemic period is another pressure that the dentists have which causes psychological distress to them^20^.

Firm and stable source of income is an absolute basic need for a person to be able to have that confidence to manage one's family needs. Seeing the pandemic period, one must keep a certain amount of money in hand for medical check-ups, precautionary measures, hospital bills, daily necessities such as food. Also depending on the availability and the changing rules and regulations one must be able to manage all those things along with their mental stability. Dentists or any other medical professional must be aware of the time-to-time changes in the guidelines or rules and regulations given by the World Health Organization or any other higher authority. So, one can adapt those measures in their respective clinics and be more cautious and alert with respect to the safety of the patients as well as themselves. Proper temperature and oxygen level checking of the patients should be done^17^. Also taking vaccination being the highest priority of all the measures the dentists have to make sure of the appointments for themselves and their family for vaccinations which also is difficult in these times where copious number of new cases are seen occurring daily. The count has made another impact of inducing stress in the minds of all the medicos as well as the common people.

## Discussion

The main factors affecting the dentists on psychological terms are anxiety, apprehension, depression, stress, concern, sadness, emotional distress, maladaptive behaviour.

### Emotional distress

It can be called suffering for an experience. Traumatizing experiences like losing your loved ones can cause emotional distress. It can show behavioural changes which mean physical and emotional reaction to a situation in a person like getting scared for no reason, self-destructive behaviour can be seen, trouble in concentration, irritability. A person might also feel hopeless for the future. Overwhelming guilt can be experienced and the affected person might feel ashamed of himself.

Dentists are like any other individuals confused, frustrated and distressed by large and growing financial losses and conflicting messages from authorities along with diminishing supplies of health care products. In addition to it, home quarantine has been an alien measure to many people facing it for the first time and is related to boredom, irritability and stigma^8^.

Extreme emotional and psychological burden is experienced by dentists as they need to balance their “duty to treat” along with their parallel duties to their family and loved ones.

Risk perception plays a very crucial role when we bring psychological impact into the conversation. It is a subjective thought that people make about risks they own on their personal well-being. Such perceptions include precautionary actions that are to be taken.

Self-efficacy refers to the belief people have in themselves to accept these preventive measures. This not only ensures their proper physical fitness but also their healthy lifestyle.

Major chunk of their thought process depends upon various personality traits that a human has such as dynamicity, susceptibility, empathy, imagination, defensiveness, introversion that can be evaluated by a GSE scale that provides a global score^12^.

During the first month of the pandemic there was no difference in level of stress between the general population and dentists. Over the period as most of the population have been home quarantined, only health workers are working at the forefront as a result level of stress gradually increased amongst this group comparatively^10^.

There have been several topics of research on covid-19 pandemic consideration of pre and post psychological issues is one of the crucial steps for which self-awareness can be a protective factor during such a crisis. In fact, increasing levels of self-awareness corresponds to decreased mental discomfort. It has been observed that females have more risk of psychological distress especially during lockdown duration while certain individuals who are either parent, are of older age tend to have lower risk comparatively.

### Maladaptive behaviour

This is a kind of behaviour which normally arises after a traumatic experience or event. It basically brings in difficulties for a person to adapt to new sudden changes. For e.g., quarantine, constantly wearing masks, constantly sanitizing hands, keeping social distance. Traumatic experiences usually make a person feel emotionally numb and that eventually leads to mood swings and anguish expressing behaviour that becomes a little bit difficult for the family members to tackle, handle and manage.

These uncertain times can produce some changes in the behaviour of the person. Being one of the riskiest groups of professionals in the pandemic era dentists do suffer behavioural problems especially when they have to balance their fear of being contaminated while treating patients.

Moreover, it's a well-known fact that poor economic conditions or uncertain times can cause psychological anxiety and restlessness in people.

Economic crisis can cause a major downfall in the living conditions. How people perceive such conditions can stimulate and transform people's mental health in a short period of time. Prevention and control policies made by the government have a significant role on the mental health of the people. Measures taken by the government generates a sense of security amongst the people and are more likely supported by the people in the country. Hence support by such government policies can be significantly correlated with the mental health of the public in the country^16^.

Thus, when fear becomes disproportionate it becomes maladaptive and harmful by being a disturbing mental health component. Public health policies can be important and dentists should consider all the measures to decrease anxiety and job insecurities among dentists^13^.

On the other hand, patients are also afraid of undergoing dental treatments due to risk of contamination. Additional funds should be allocated to create awareness among dentists.

Pandemics are responsible for starting fearful reactions and anxiety. Anxieties can sometimes lead to irrational fear of interpreting harmless bodily sensation as evidence of Covid-19. This can happen to patients as well as dentists. But taking dentists in consideration, these irrational fears can lead to behavioural charges^14^.

Adding fuel to the problems, normal adaptive coping techniques such as exercises, leisure time, social meetings and sports are all restricted which are leading to uncertainty, boredom and isolation^15^.

Successful public health outcomes largely depend on the healthcare workers like dentists and they can really affect psychologically thus affecting their overall performance in clinics.

### Existential Study

Legitimate fears like concern for family, health, financial stability and despite usage of protective measures are often present. In addition to this study, the hypothesis or addition to the dentists also puts light on dental staffs that are in the same situation as dentists.

A German study says that dentists working in a university might experience distress compared to a clinical practitioner^3^. The reason for this is assumed that the time divided for a university dentist is equal for education, research and patient treatment while for clinical practitioner it's completely patient treatment. It is presumed that younger dentists can sustain the impact of Covid-19 virus in a better fashion as compared to other dentists. An area of most stressful situations to look after is dentists who are attending treatment for Covid-19 patients in quarantine centres.

People who are affected by systemic diseases are told to take exceptional care which brings into notice that there might be older people at the dentist's home which is another reason for psychological distress. So many questions like what would happen if unknowingly the dentist treats a patient and the patient turns out to be a potential carrier arise. For such fears, the dentists are supposed to follow all the rules and regulations provided by the officials. The infection protocols, the cross-infection protocols play a particularly significant role during such times and one must abide by them to ensure one's safety and wellness. Thus, the appointed front line and health line workers are truly under tremendous pressure and anxiety in this pandemic period. The people will always be grateful for their remarkable contribution to the community. This pandemic has caused an absolute burnout amongst all the dentists. However, analysing the current count of death rate it might really be traumatic for them and obvious for them to have such fears and distress. This might also result in post stress symptoms post pandemic period and which might affect their health drastically^20^.

There is also a terminology called institutional betrayal that comes into the scenario. The institutions might not assure the employment opportunities to new dentists and restriction under pressure of the higher authorities can be done. The level of safety provided and assured in the institutions is a thing to bother. The inadequacy or shortage of PPE kits adds on to it^19^. Financial stress factor stays on the heap of all the other factors.

The patients who are suffering from critical conditions or co morbidities tend to cause an increase in the count of the patients with hospital complications and mortality rate 3. Females of age more than 50 years tend to show immune deficiency hence contribute even more to the increase in the count of the patients who are prone to the covid-19 infection as a result, causing more of psychological distress and anxiety. Also, in such situations the dentists who are affected by a systemic disease or are undergoing a treatment for the same will obviously have no confidence to run a clinic with the courage of not getting infected.

The mental wellbeing of dentists should be a vital point to be noted for the safety and vulnerability of the profession in present as well as in future. As much as a patient's health and mental state is important the same, in fact more weight should be given to the mental health of the dental practitioners who are treating them and helping them to have a better smile and enabling them to be a better version of themselves.

### Measures

Use of antibacterial mouthwashes before initiation of any treatment is recommended^20^. Reports have said that most of the dentists ignore this part of the precautionary measures which should be prioritised the most. During the times of such an infectious outbreak such kind of precautionary measures cannot be ignored. The severity and mortality rate due to this virus cannot be ignored at any cost.

Continuous wear of masks, protective shields kits while doing patients have saved the dentists or any other medical professional till now^14^.Nowadays patients when entering the dental clinic are given foot covers to wear to avoid the slightest chance of the patient causing infection. To avoid spread of aerosols high volume suction can be used. WHO recommends frequent washing of hands with soap and water to avoid infection or use of alcohol-based sanitizer as a precautionary measure. Even the slightest of negligence can cause the person to get infected and as doctors too they are setting an example for all of us.

Dentists being the most susceptible medical professionals should be given the highest priority for vaccination as well as other precautionary measures that are to be adapted should be improvised considering the risk factor in the profession of dentistry. This will not only ease out the psychological fear, stress, anxiety and apprehension from their minds but also help them to work better for the welfare of the people considering their safety and health first.

Donning and doffing are another two procedures that are to be done with great care and alertness. More strict and preventive measures are expected to be made in future as the seriousness amongst the people in the country increases. Nowadays tele-dentistry is another mode of easy treatment choice to avoid contact. The dentists can opt for this way and help people to assess their own problems thus avoiding the contact between the dentist and the patients and only the emergency cases can be called in the clinic that too on an appointment basis. They can get a pre scheduled confirmation of the appointment from the patient^19^. To reduce psychological impact in the patients too, the online face to face therapy or psychotherapy can be opted for. This will lead to exchange of ideas amongst the people and the doctors and raise a feeling of awareness and caution. Behavioural therapy can be used which teaches you several relaxation techniques and due to which psychological pressure can be lowered and the mental state of the doctors can be taken care of.

Moreover, presence of co morbid factors like blood pressure, diabetes, cardiac abnormalities and epilepsy can further complicate the dental procedures in these cases 7. Toothaches being one of the most painful diseases cannot be left untouched without a doctor handling the situation. There are several oral manifestations caused that are being observed in several cases affected from the SARS Covid virus. SARS CoV-2 can infect oral cavity by causing superficial necrosis and soft tissue oral ulcerations. As we are aware that oral mucosa or oral cavity is the main route of infection for a person to catch the novel corona virus infection, it binds to the Angiotensin converting enzyme (ACE2) receptor that is found in epithelial cells of tongue, salivary glands and cell membrane of various tissues thus explaining the loss of smell and taste sensation^22^. It is also capable of causing cellular and tissue level haemorrhage which can further cause haemorrhagic and stomatitis like lesions. Perivascular lymphocytic infiltration is the subject of dental interest in cases with SARS Covid infection. There are several cases that are being reported with vesiculobullous and macular lesions. Thus, this specific virus can be a sole reason in the onset of oral diseases^23^.

With over millions of cases in the country and globally the morals and ethics of a dentist doesn't allow them to refuse any patient for treatment. Therefore, the chances of Covid-19 transmission from patients to the dentist can increase. Unlike any other profession like IT professionals, teachers, a dental professional is one of the most critical professionals in these times and thus there is a need to access their psychological states often. As this might not just only impact dentists but hundreds, thousands and potentially millions of potentially dental students which might require doctors of this specialty for reference and guidance will remain devoid of it. Dentists are amongst the most susceptible professionals and need to get vaccination doses primarily.

In India Covi Shield and Covaxin and in other parts of the world Moderna Covid-19 vaccine, Pfizer, Johnson and Johnson, Sputnik can be conveniently used. Dentists themselves should also be conscious enough about their psychological impact. Psycho educational interventions and behavioural strategies can be practised in such times^12^. Meditation, biofeedback, exercises and having strong emotional support from loved ones can help a professional cope in these times.

## Conclusion

Although dental science today has reached to a level of perfection for treatment planning in dentistry, not much work has been done in educating dentists about how to cope up with stress especially in a pandemic situation like this. With growing cases day by day how dentists across the globe have changed this practice and have adopted the new change e.g.: right from only doing emergency cases to being selective towards the patients. In such a scenario universal precautions and PPE kit works best adhering to WHO protocols and usage of the resources such as Arogya Setu application especially in India to their optimum level is required. Dentists need to align as per government and DCI guidelines. Any non-emergency case patient approaches and treatment is not possible then basic medical check-up and meticulous case history is required. In a pandemic where the most important and crucial defence mechanism for Covid-19 protection is being used, dentistry is a field in which professionals have to dig deep down in the oral cavity of patients and work for hours. Hence surely dentists are no exception to fear, psychological distress and financial disruption of income if not more. This isn't just about the present scenario but possible outcomes on the global dental industry too which can take a huge toll on average dentists not just in India but globally.
